# Development of the First Health-Related Quality of Life Questionnaires in Arabic for Women With Polycystic Ovary Syndrome (Part III): Scoring System Interpretation

**DOI:** 10.7759/cureus.32166

**Published:** 2022-12-03

**Authors:** Samih A Odhaib, Mahmood Thamer Altemimi, Husam J Imran

**Affiliations:** 1 Adult Endocrinology, Thi Qar Specialized Diabetes Endocrine and Metabolism Center (TDEMC), Thi Qar, IRQ; 2 Endocrinology, Thi Qar Specialized Diabetes Endocrine and Metabolism Center (TDEMC), Thi Qar, IRQ; 3 Adult Endocrinology, Maisan Specialized Diabetes, Endocrine and Metabolism Center, Maisan, IRQ

**Keywords:** arabic, questionnaires, quality of life (qol), polycystic ovary syndrome (pcos), iraq

## Abstract

Background: Our team developed the first highly reliable, validated, easily comprehensible, and self-administered polycystic ovary syndrome quality of life (PCOSQOL)-42 and PCOSQOL-47 questionnaires for unmarried and married women with polycystic ovary syndrome (PCOS), respectively. Using such scales needs a scoring system that covers the responses to each item per domain and overcomes the missing responses. we developed a scoring system for PCOSQOL-42 and PCOSQOL-47 to interpret the items' responses at any similar population.

Methods: The scoring was inspired by the 5-point Likert scale that was used during the creation of PCOSQOL-42 and PCOSQOL-47, where each item represents the woman's experience in the last two weeks before seeking consultation, i.e., Never=5 or no effect, on the health-related quality of life (HRQOL), (Seldom=4), (Quite often=3), (Very often=2), and (Always=1 or the maximal effect on the QOL). The sum of the total points in each item per domain was evaluated. Then we divided the results by the number of the items that had been scored only to get the final domain score as a (mean ± SD). The ultimate or final score per the questionnaire was gained from the sum of individual domain scores divided by the number of domains that had been evaluated. Ultimately, the first interval values (from 1 to < 3 points) represent marked effects on HRQOL; second interval values (from 3 to < 4 points) represent the marginal effect on HRQOL; third interval values (from 4 to < 5 points) represent the minimal effect on HRQOL; fourth interval (5 points) represents no effect on HRQOL.

Results: The lower the score, the greater the negative impact on HRQOL. Although all domains in both questionnaires showed a marked reduction in HRQOL, women in either cohort were more concerned with their body image dissatisfaction and psychological distress induced by PCOS than their reproductive concerns. The mean score calculated for the questionnaire had a greater negative impact than the emotions subscale and was similar to the subscale for infertility. All mean points per item and domain values indicate a marked effect (< 3 points) on QOL using PCOSQOL-42 and PCOSQOL-47. All values referred to a marked reduction in PCOSQOL-47 points, whether item or domain-wise.

Conclusions: The current scoring system provides an easy way to interpret the responses in both questionnaires and overcome the missing responses in any item per domain. There was a marked impact on all domains of HRQOL using both questionnaires, with a special impact on body image and psychological concerns. The responses of women in either cohort to the sexual and reproductive items were extremely high, reflecting the scope of this problem in the life of women with PCOS.

## Introduction

The highly reliable, highly validated, easily comprehensible, and self-administered polycystic ovary syndrome quality of life (PCOSQOL)-42 and PCOSQOL-47 questionnaires for unmarried and married women with polycystic ovary syndrome (PCOS), respectively, are the first health-related quality of life scales that account for the difference in the responses of those women with PCOS, specifically with regard to sexual activity of married women in some communities and cultures [1,2]. Our previous work on this topic showed the initial three phases of creating and modifying these two scales. In this article, we show the methods of scoring and the initial scoring results of the enrolled women in the final phase III of the study (validation phase) in which the final drafts of the PCOSQOL-42 and PCOSQOL-47 were developed and used for unmarried and married women with PCOS, respectively.

## Materials and methods

The enrollment details for the women with PCOS were discussed in our previous work for PCOSQOL-42 and PCOSQOL-47 questionnaires [1,2]. The responses of women with PCOS who were enrolled in the validation phase of PCOSQOL-42 and PCOSQOL-47 were further evaluated to measure the exact effects of the PCOS status on the health-related quality of life (HRQOL) domains of the affected women using the questionnaires above.

The response to each item is made using the five-point Likert scale where (Never=5 or no effect on the HRQOL), (Seldom=4), (Quite often=3), (Very often=2), and (Always=1 or the maximal effect on the HRQOL). Women in both cohorts recorded their responses to the variable items in different domains of both questionnaires according to the presumed effect on their HRQOL. Women were free to respond to or decline any item in any domain.

The sum of the total points in each item per domain was evaluated. Then we divided the result by the number of items that had been scored only to get the final domain score as a mean ± standard deviation (SD). The ultimate or final score per the questionnaire was gained from the sum of individual domain scores divided by the number of domains that had been evaluated.

For both questionnaires, the interpretation of the domain score points, or the final questionnaire points, was done according to the following ranges: first interval values (from 1 to < 3 points) represent marked effects on HRQOL; second interval values (from 3 to < 4 points) represent the marginal effect on HRQOL; third interval values (from 4 to < 5 points) represent the minimal effect on HRQOL; fourth interval (5 points) represents no effect on HRQOL.

The normal distribution of the score points per item or domain could not be ensured even with the logarithmic transformation of the score points.

All the study phases were per the 1964 Declaration of Helsinki and its later amendments or comparable ethical standards and with the ethical committee and the institutional review board (IRB) standards of Faiha Specialized Diabetes Endocrine and Metabolism Center (FDEMC) from whom ethical approval (E43/3/2018) was obtained for this study. All enrolled women signed informed consent in Arabic before participating in the study.

## Results

The final drafts of the PCOSQOL-42 and PCOSQOL-47 and the general characteristics of the enrolled women in both cohorts are discussed in our previous work [2].

The lower the score, the greater the negative impact on HRQOL. Although all domains in both questionnaires showed marked reduction in HRQOL, women in either cohort were more concerned with their body image dissatisfaction and psychological distress induced by PCOS than their reproductive concerns. The mean score calculated for the total PCOSQ had a greater negative impact than the emotions subscale and was similar to the subscale for infertility.

Table 1 and Table 2 show variable responses to different items in different domains.

**Table 1:** Results of 362 unmarried women using PCOSQOL-42 The range of responses to all items is rated from 1 to 5. PCOS: Polycystic ovary syndrome, SD: Standard deviation, PCOSQOL: Polycystic ovary syndrome quality of life

PCOSQOL-42 Domains	In the previous two weeks, have you…	No. of Respondents n (%)	Mean Points per Item (SD)	Mean of Responses per Domain (SD)	Effect on the PCOSQOL-42				No Response
					Marked	Marginal	Minimal	No Effect	
Psychological and Emotional Status Domain	A1: Suffered from bad mood due to PCOS?	357 (98.62)	2.13 (1.10)	2.45 (0.97)	249 (68.79)	81 (22.38)	29 (8.0)	2 (0.55)	1 (0.28)
	A2: Felt pessimistic about the treatment?	356 (98.34)	2.26 (1.19)						
	A3: Felt the urge to abandon treatments because of repetitive visits to doctors?	349 (96.41)	2.34 (1.24)						
	A4: Felt frequent tantrums due to PCOS?	353 (97.51)	2.65 (1.48)						
	A5: Experienced fear of diseases such as diabetes, hypertension, and heart disease?	349 (96.41)	2.82 (1.53)						
	A6: Experienced trouble dealing with others?	356 (98.34)	2.39 (1.37)						
	A7: Suffered from low self-esteem due to PCOS?	355 (98.07)	2.44 (1.35)						
	A8: Blamed yourself for having PCOS?	353 (97.51)	2.54 (1.35)						
	Total Domain A	333 (91.99)							
Menstrual Disorders and Fertility Domain	B1: Felt the need to decrease your weight to control PCOS?	359 (99.17)	2.12 (1.20)	2.52 (1.00)	255 (70.4)	56 (15.5)	44 (12.2)	7 (1.9)	0
	B2: Felt concerned about future infertility?	357 (98.61)	2.46 (1.42)						
	B3: Felt concerned about the complete cessation of menstruation?	353 (97.51)	2.84 (1.60)						
	B4: Felt concerned about menstruation at long intervals?	357 (98.61)	2.60 (1.38)						
	B5: Felt a regular need for oral contraceptive pills to control PCOS?	336 (92.82)	2.67 (1.46)						
	B6: Felt you would accept all other PCOS manifestations if assured of pregnancy?	351 (96.96)	2.57 (1.45)						
	B7: Experienced feelings of fear of cancer due to PCOS?	360 (99.45)	2.32 (1.28)						
	Total Domain B	328 (90.61)							
Body Image Domain	C1: Dissatisfied with some aspects of your appearance?	361 (99.72)	2.19 (1.06)	2.42 (1.00)	263 (72.7)	54 (14.9)	38 (10.5)	7 (1.9)	0
	C2: Tried to hide some flaws in your appearance?	361 (99.72)	2.05 (1.15)						
	C3: Spent a significant amount of time checking your appearance in the mirror?	356 (98.34)	2.85 (1.48)						
	C4: Felt ashamed of some part of your body?	362 (100)	2.57 (1.40)						
	C5: Fear that others will discover flaws in your appearance?	359 (99.17)	2.41 (1.30)						
	C6: Feel others are speaking negatively about your appearance?	362 (100)	2.37 (1.28)						
	C7: Avoided looking at your appearance in the mirror?	362 (100)	2.48 (1.26)						
	Total Domain C	351 (96.96)							
Hair Disorders and Acne Domain	D1: Felt embarrassed about having excess facial and body hair?	362 (100)	1.86 (0.95)	2.17 (0.74)	299 (82.6)	53 (14.6)	10 (2.8)	0	0
	D2: Felt that alopecia is disturbing your appearance?	362 (100)	1.77 (0.92)						
	D3: Been concerned about the progression pattern of excess body and facial hair?	362 (100)	1.79 (0.97)						
	D4: Felt that alopecia leads to a decrease in your attraction and femininity?	360 (99.45)	2.13 (1.29)						
	D5: Felt concerned about rapid re-growth of unwanted hair after its removal?	360 (99.45)	2.33 (1.32)						
	D6: Always worn a headscarf to cover your head due to alopecia?	362 (100)	1.99 (1.04)						
	D7: Fear of facial acne leaving permanent scars?	360 (99.45)	2.14 (1.12)						
	D8: Felt that acne is disturbing your appearance?	359 (99.17)	2.60 (1.62)						
	D9: Felt that treatment of alopecia needs a long time and is worthless?	357 (98.61)	2.24 (1.32)						
	D10: Felt the need to cover your body and face because of excess hair?	347 (95.86)	2.82 (1.58)						
	D11: Avoided social circumstances due to alopecia?	353 (97.51)	2.15 (1.21)						
	Total Domain D	332 (91.71)							
Coping Domain	E1: Tried to consult a medical expert about what you think is a flaw in your appearance?	348 (96.13)	2.77 (1.57)	2.61 (1.15)	236 (65.2)	58 (16.0)	60 (16.6)	8 (2.2)	0
	E2: Embarrassed to engage in social activities because of your appearance?	353 (97.51)	2.74 (1.58)						
	E3: Compared your appearance with other women who you think are more physically attractive than you?	352 (97.24)	2.50 (1.47)						
	E4: Felt disappointed about the cure?	347 (95.85)	2.84 (1.66)						
	E5: Avoided social circumstances due to excess body hair	352 (97.24)	2.56 (1.41)						
	E6: Felt a lack of family support and acceptance of your disease?	362 (100)	2.64 (1.46)						
	E7: Felt difficulty in communicating with other women who have PCOS?	362 (100)	2.46 (1.41)						
	E8: Felt a lack of satisfaction with being a woman?	362 (100)	2.40 (1.28)						
	E9: Concerned about your future role as a wife?	360 (99.45	2.59 (1.34)						
	Total Domain E	325 (89.78)							
Overall PCOSQOL-42 Domains				2.43 (0.83)	260 (71.8)	84 (23.2)	18 (5.0)	0	0

**Table 2:** Results of 406 unmarried women using PCOSQOL-47 The range of responses to all items is rated from 1 to 5. PCOS: Polycystic ovary syndrome, SD: Standard deviation, PCOSQOL: Polycystic ovary syndrome quality of life

PCOSQOL-47 Domains	Items (number of respondents)	No. of Respondents n (%)	Mean points per Item (SD)	Mean of responses per domain (SD)	Effect on the PCOSQOL-47				No Response
					Marked	Marginal	Minimal	No Effect	
	In the previous two weeks, have you...								
Psychological and Emotional Status Domain	A1: Suffered from a bad mood due to PCOS?	396 (97.54)	2.14 (1.22)	2.38 (0.91)	311 (76.6)	64 (15.8)	31 (7.6)	0	0
	A2: Felt easily tired?	396 (97.54)	2.28 (1.34)						
	A3: Felt pessimistic about the treatment?	402 (99.01)	2.47 (1.33)						
	A4: Felt the urge to abandon treatments because of repetitive visits to doctors?	382 (94.09)	2.72 (1.60)						
	A5: Felt frequent tantrums due to PCOS?	375 (92.36)	2.72 (1.62)						
	A6: Experienced trouble dealing with others?	381 (93.84)	2.36 (1.38)						
	A7: Blamed yourself for having PCOS?	390 (96.06)	2.07 (1.14)						
	A8: Experienced fear of diseases such as diabetes, hypertension, and heart disease?	392 (96.55)	2.18 (1.41)						
	A9: Suffered from low self-esteem due to PCOS?	391 (96.31)	2.32 (1.26)						
	Total Domain A	370 (91.13)							
Fertility and Sexual Life Domain	B1: Felt fear of abortion?	364 (89.66)	2.27 (1.50)	2.45 (1.10)	280 (69.0)	63 (15.5)	58 (14.3)	0	5 (1.2)
	B2: Felt uselessness of sexual intercourse due to infertility?	365 (89.90)	2.26 (1.59)						
	B3: Felt sad seeing pregnant women?	371 (91.38)	2.21 (1.32)						
	B4: Experienced concern about future infertility?	356 (87.68)	2.51 (1.52)						
	B5: Experienced fear of divorce or separation?	366 (90.15)	2.66 (1.41)						
	B6: Felt sad seeing children?	355 (87.44)	2.69 (1.36)						
	B7: Felt a lack of sexual desire?	364 (89.66)	2.65 (1.51)						
	B8: Felt ashamed of sexual coldness/unresponsiveness?	379 (93.35)	2.53 (1.33)						
	B9: Felt unsatisfied with sexual life?	373 (91.87)	2.36 (1.28)						
	B10: Experienced a lack of orgasm?	382 (94.09)	2.41 (1.36)						
	Total Domain B	282 (69.46)							
Body Image Domain	C1: Tried to consult a medical expert about what you think is a flaw in your appearance?	398 (98.03)	2.30 (1.19)	2.54 (1.00)	227 (68.23)	77 (18.96)	48 (11.82)	0	4 (0.99)
	C2: Dissatisfied with some aspects of your appearance?	396 (97.54)	2.10 (1.16)						
	C3: Tried to hide some flaws in your appearance?	398 (98.03)	2.98 (1.48)						
	C4: Experienced fear of treatment complications?	395 (97.29)	2.68 (1.40)						
	C5: Ashamed of some part of your body?	398 (98.03)	2.54 (1.47)						
	C6: Spent a significant amount of time checking your appearance in the mirror?	402 (99.01)	2.37 (1.26)						
	C7: Embarrassed to engage in social activities because of your appearance?	396 (97.54)	2.69 (1.45)						
	C8: Felt others are speaking negatively about your appearance?	399 (98.28)	2.48 (1.27)						
	C9: Compared your appearance with other women who you think are more physically attractive than you?	399 (98.28)	2.61 (1.43)						
	C10: Fear that others will discover flaws in your appearance?	396 (97.54)	2.57 (1.31)						
	C11: Avoided looking at your appearance in the mirror?	356 (87.68)	2.48 (1.21)						
	Total Domain C	348 (85.71)							
Hair Disorders and Acne Domain	D1: Felt concerned about rapid re-growth of unwanted hair after its removal?	402 (99.01)	1.87 (1.00)	2.23 (0.80)	334 (82.26)	47 (11.58)	21 (5.17)	0	4 (0.99)
	D2: Felt concerned about the progression pattern of excess body and facial hair?	402 (99.01)	1.92 (0.98)						
	D3: Felt that acne is disturbing your appearance?	402 (99.01)	1.83 (0.92)						
	D4: Felt embarrassed about having excess facial and body hair?	402 (99.01)	2.13 (1.13)						
	D5: Felt that alopecia leads a decrease in your attraction and femininity?	401 (98.77)	2.54 (1.44)						
	D6: Felt that alopecia is disturbing your appearance?	395 (97.29)	2.52 (1.37)						
	D7: Felt the need to cover your body and face because of excess hair?	400 (98.52)	2.24 (1.13)						
	D8: Felt that treatment of alopecia needs a long time and is worthless?	396 (97.54)	2.20 (1.22)						
	D9: Fear of facial acne leaving permanent scars?	401 (98.77)	2.52 (1.57)						
	D10: Avoided social circumstances due to excess body hair?	384 (94.58)	2.18 (1.30)						
	D11: Always worn a headscarf or a veil to cover your hair due to alopecia?	376 (92.16)	2.52 (1.35)						
	Total Domain D	366 (90.15)							
Obesity and Menstrual Disorders Domain	E1: Felt concerned about a fast return to your previous weight after any weight loss?	388 (95.57)	2.24 (1.19)	2.44 (0.91)	311 (76.60)	58 (14.28)	33 (8.13)	0	4 (0.99)
	E2: Felt concerned about the complete cessation of menstruation?	390 (96.06)	2.12 (1.07)						
	E3: Felt the need for regular oral contraceptive pills to control PCOS?	365 (89.90)	2.78 (1.37)						
	E4: Felt you would accept all other PCOS manifestations if assured of pregnancy?	382 (94.09)	2.62 (1.57)						
	E5: Experienced feelings of fear of cancer due to PCOS?	351 (86.45)	2.05 (1.27)						
	E6: Felt a lack of satisfaction with your current role as a wife?	377 (92.86)	2.45 (1.48)						
	Total Domain E	303 (74.63)							
Overall PCOSQOL-47 Domains				2.42 (0.80)	321 (79.06)	58 (14.29)	27 (6.65)	0	

All mean points per item and domain values indicate a marked effect (< 3 points) on HRQOL using PCOSQOL-42 and PCOSQOL-47. The response rate in PCOSQOL-42 is more than 90% for all domains with many items showing a 100% response rate. While the response rate for PCOSQOL-47 ranges from 69% (n=282) in the fertility and sexual life domain to 91% (n=370) in the psychological and emotional status domain.

In Table 1, which demonstrates the response of 362 unmarried women to the PCOSQOL-42, about 72 % of unmarried women with PCOS (n=260) expressed a marked reduction in the PCOSQOL-42, with a mean value (2.43 ± 0.83 points) i.e., less than three points. More effects were seen in the morphological domains (hair, acne, and body image domains) rather than the psychological, emotional, and coping domains regarding the mean values. However, all values referred to a marked reduction in PCOSQOL-42 points.

A reversed effect pattern was observed in (Table 2) which demonstrates the response of 406 married women to the PCOSQOL-47, in which 79% of those women (n=321) expressed a marked reduction in the PCOSQOL-47, with a mean value (2.42 ± 0.80 points). The married women in this cohort were more concerned with their PCOS-related psychological and emotional issues relatively more than their morphological features. Again, all values referred to a marked reduction in PCOSQOL-47 points, whether item- or domain-wise.

Although around 70% of women in PCOSQOL-42 (n=255) and PCOSQOL-47 (n=280) described a marked reduction in the items regarding their PCOS-related fertility concerns, the current or future fertility was not their ultimate or highest priority.

## Discussion

The PCOSQoL-42 and PCOSQoL-47 are the first assessment tools that have properly addressed the concept of being sexually active and inactive by avoiding the inclusion of sexual themes in the scale described for unmarried women, making these two questionnaires applicable for any community with similar sexual norms. That is why the study findings are novel and may represent a new dimension in the HRQOL estimation in women with PCOS. This syndrome is highly destructive to the 'feminine' identity.

The majority of enrolled women in both cohorts had various negative impacts on different domains of their HRQOL, which was reflected by the reduction in the corresponding score of each item per domain, the whole domain, and consequently the total score per each questionnaire.

Given the effect PCOS has on women's physical health and emotional well-being, it is not surprising that the highest effect of PCOS symptoms on HRQOL impairment is exerted by self-esteem, body image, and relative sexual function.

The effects of PCOS on the HRQOL of the affected women influence their psychological and physical well-being. Polycystic ovary syndrome may trigger psycho-social problems, mental distress, sexual and marital problems, loss of control, and lowered self-esteem [3-5]. And these psychiatric illnesses may go undetected [6].

Women with dysmorphic features like hirsutism as a part of clinical hyperandrogenism reported more serious concerns using our scale items. Previous studies revealed that women with hirsutism experience dramatic reduction in emotions domain, including feeling moody, worried, depressed, and having low self-esteem due to PCOS due to social phobia or anxiety-evoking situations [7-10]. Most hirsute women spend considerable time and energy attempting to control facial hair, attention to appearance, which appears to facilitate better adjustment [8].

As the first scale to be used to measure the HRQOL in women with PCOS, which dealt with sexual items in the Iraqi population, we get different important findings. Unexpectedly, the questions about sexual function and fertility received a high response rate among married women with PCOS (87.4% to 93.4%) with markedly low scores, which reflects the scope of this hidden problem in those women. The study revealed a marked fear of current and future infertility in both questionnaires, similar to the results of PCOSQ-50 by Amiri et al. [11].

The gender-related role, loss of feminine characteristics, marked clinical hyperandrogenism, and disruption in marital relationships markedly affected the existence of self-perceived womanhood or femininity, as seen in many items in different domains of PCOSQOL-42 and PCOSQOL-47.

Different quantitative and qualitative studies showed that women with various features of clinical hyperandrogenism or different dysmorphic changes due to PCOS may have distorted body image of themselves, feel less feminine or lack self-perceived physical attractiveness, with severe emotional and psychological distress, and negative impact on HRQOL, which led to low self-esteem and social interaction among them [4,5,12-16]. Both Amiri et al. and McCook et al. consider weight problems as the main feature in women with PCOS, which had a negative impact on the HRQOL of women with PCOS [5,7]. They ranked other effects after the weight problems.

For infertile women, the psychological burden is often associated with divorce, low social status, and lowered self-perception because motherhood is perceived as an important part of female identity. In some cultures, motherhood is the only way for women to enhance their status in their families and community [8].

The fear of current or future infertility negatively impacted the HRQOL of women with PCOS. These results were similar to the Bazarganipour et al. study, which revealed that the extent of infertility on HRQOL varies according to sociocultural factors, traditions, and religious beliefs in a cohort of Iranian women with PCOS [17]. On the contrary, the effect of infertility on negative psychological consequences and somatic complaints reported by women with PCOS was negated by McCook et al.; they studied a comparable cohort of American women with PCOS [7]. Such differences are valid given the similar sociodemographic characteristics of the Iranian cohort and ours and the difference with the American cohort.

Many women in this study experienced variable sorts of sexual frustration with different degrees of infertility fear. The nature of this study is observational and cross-sectional, which made us unable to determine any causal relations between the items. The exact direct and indirect causal mechanisms underlying the association between psychosexual variables and HRQOL have not been well understood and appreciated.

The broad symptomatic spectrum of women with PCOS may affect their sexual function directly or indirectly by influencing their feminine identity and disturbed aesthetic standards through obesity and body image dissatisfaction, psychological distress, and social maladjustment, frequently perceived by women with PCOS [4,5,18]. On the other hand, menstrual irregularities in women with PCOS negatively impact HRQOL due to the feeling of less femininity and its marked effect on many aspects of their reproductive and general health [7,8,12,15]. Hahn et al. reported that women with PCOS believed that their sexual partners were less satis­fied with their sexual lives and considered themselves unattractive [18,19].

As the first scale to evaluate the sexual item for married women, we did not have comparable items in other scales. We did not expect such a high response rate for intimacy items in the sexual domains. Items regarding orgasm and sexual satisfaction received very low scores, which reflected their impact on the marital relations of women with PCOS.

Hahn et al. showed that the incidence of sexual thoughts and fantasies and subsequent arousal was negatively correlated to body mass index (BMI) [19]; however, orgasm frequency was not affected in those women regardless of their BMI [20]. Women with PCOS may have decreased sexual attractiveness, satisfaction, and self-worth, which seems to be influenced by both endocrine and psycho-social factors [18-21].

The PCOSQOL-42 and PCOSQOL-47 are the first scales that contained highly valid and reliable items dealing directly with the effect of acne as a dysmorphic feature on the HRQOL of women with PCOS [1,2]. Acne is an important sign frequently overlooked or neglected by the previous questionnaires [11,22]. A very important finding is the response rate to items that deal with the dysmorphic effect of acne, which was more than 98%, which highlighted the impact of this feature on the HRQOL of women with PCOS in both cohorts.

We also found that women with PCOS in both cohorts expressed marked fear of future metabolic, cardiovascular, and malignant diseases as complications for their current PCOS. Research indicates PCOS is a risk factor for endometrial cancer and metabolic disturbances [3,23].

Another important finding in this study was that we could not demonstrate why respondents to items A3, D9, E1, and E6 in PCOSQOL-42 and items C1 and C4 in PCOSQOL-47 gave such markedly low scores for these items which deal with satisfaction and belief in their current therapeutic regimens, and the psych-social support from their family, healthcare personnel, and community support. Previous sporadic studies evaluated such frustration over lack of support, which concentrates on the poor healthcare professional response to the reality or genuineness of signs and symptoms and the need for confirmatory tests over the clinical decision [15,24]. The frustration about familial support, effective therapeutic interventions, future solutions, and perceived or future fertility may give us a snapshot of the coping mechanism which could be dealt with by these women. The coping items scored markedly low, reflects the scope of this health problem.

The study had several limitations, the most important being that these questionnaires were developed in Iraq and may reflect only the status of women with PCOS who speak the Arabic language in the multilinguistic Iraqi population. Another limitation is the referral bias of these women to these tertiary centers, which are highly specialized in dealing with such conditions. The recall bias may influence the responses of the referred women. All the reported data were self-reported, and the possibility of inaccuracy could not be ruled out. Additionally, no conclusions can be drawn regarding causality due to the nature of the study.

## Conclusions

All domains of HRQOL of women with PCOS are affected regardless of their marital status. There was a marked reduction in scores for items concerning body image misperception and dissatisfaction. Women in both cohorts expressed a marked impact on femininity and the feeling of womanhood. All the changes in the HRQOL of these women in either cohort culminated in psychosexual disturbances and life dissatisfaction.

Multi-center, longitudinal, and follow-up studies are needed to show the effect of therapeutic interventions on different domains of PCOSQOL-42 and PCOSQOL-47. Given that these scales have never been used in other languages, a well-designed questionnaire could be used in similar societies with similar practices after making culture-specific adjustments.
